# The effects of blurred visual inputs with different levels on the cerebral activity during free level walking

**DOI:** 10.3389/fnins.2023.1151799

**Published:** 2023-04-17

**Authors:** Mingxin Ao, Shuang Ren, Yuanyuan Yu, Hongshi Huang, Xin Miao, Yingfang Ao, Wei Wang

**Affiliations:** ^1^Department of Ophthalmology, Peking University Third Hospital, Beijing, China; ^2^Beijing Key Laboratory of Restoration of Damaged Ocular Nerve, Peking University Third Hospital, Beijing, China; ^3^Department of Sports Medicine, Institute of Sports Medicine of Peking University, Peking University Third Hospital, Beijing, China; ^4^Beijing Key Laboratory of Sports Injuries, Peking University Third Hospital, Beijing, China

**Keywords:** electroencephalography, sensorimotor integration, motor control, mobile brain/body imaging, locomotion, adaptive generalization, visual working memory

## Abstract

**Objective:**

The aim of this study was to evaluate the effects of blurred vision on electrocortical activities at different levels during walking.

**Materials and methods:**

A total of 22 healthy volunteers (all men; mean age: 24.4 ± 3.9 years) underwent an electroencephalography (EEG) test synchronous with free level walking. Visual status was simulated by goggles covered by the occlusion foil targeted at a Snellen visual acuity of 20/60 (V0.3), 20/200 (V0.1), and light perception (V0). At each of these conditions, the participants completed barefoot walking for five blocks of 10 m. The EEG signals were recorded by a wireless EEG system with electrodes of interest, namely, Cz, Pz, Oz, O1, and O2. The gait performances were assessed by the Vicon system.

**Results:**

During walking with normal vision (V1.0), there were cerebral activities related to visual processing, characterized as higher spectral power of delta (Oz and O2 vs. Cz, Pz, and O1, *p* ≤ 0.033) and theta (Oz vs. Cz and O1, *p* = 0.044) bands in occipital regions. Moderately blurred vision (V0.3) would attenuate the predominance of delta- and theta-band activities at Oz and O2, respectively. At the statuses of V0.1 and V0, the higher power of delta (at V0.1 and V0, Oz, and O2 vs. Cz, Pz, and O1, *p* ≤ 0.047) and theta bands (at V0.1, Oz vs. Cz, *p* = 0.010; at V0, Oz vs. Cz, Pz, and O1, *p* ≤ 0.016) emerged again. The cautious gait pattern, characterized by a decrease in gait speed (*p* < 0.001), a greater amplitude of deviation from the right ahead (*p* < 0.001), a prolonged stance time (*p* = 0.001), a restricted range of motion in the hip on the right side (*p* ≤ 0.010), and an increased knee flexion during stance on the left side (*p* = 0.014), was only detected at the status of V0. The power of the alpha band at the status of V0 was higher than that at V1.0, V0.3, and V0.1 (*p* ≤ 0.011).

**Conclusion:**

Mildly blurred visual inputs would elicit generalization of low-frequency band activity during walking. In circumstance to no effective visual input, locomotor navigation would rely on cerebral activity related to visual working memory. The threshold to trigger the shift might be the visual status that is as blurred as the level of Snellen visual acuity of 20/200.

## 1. Introduction

The visual system provides crucial information about the actual changes in the planned path, which aids the individuals to adjust their walking strategy and adapt to the environment (Rietdyk and Rhea, 2006). In patients suffering from long-term loss of vision for eye diseases, a slower walking speed, shorter stride length, less trunk flexion, earlier plantar–foot contact at heel strike, and prolonged duration of stance have been detected (Nakamura, 1997; Hallemans et al., 2010). When the sighted individuals were blindfolded, they would exhibit a slower walking speed and limited movements of the hip and the ankle in the sagittal plane in comparison to the condition of normal vision (Hallemans et al., 2010). Additionally, the interference of blurred vision on gait performance has also been investigated, revealing a significant decrease in walking speed in those circumstances that simulated low vision with visual acuity of the counting finger (Ayaki et al., 2015). These recent studies suggested that visual inputs played an important role in locomotor navigation. To investigate the mechanism underlying these effects of visual inputs on walking performance, the sensorimotor integration related to vision and the cerebral dynamics synchronous with walking at different visual statuses should be analyzed intensively.

Scalp electroencephalography (EEG) is a valuable method in the investigation of cerebral activity during walking. Previous studies have confirmed that virtual reality with perturbations during treadmill walking would induce increased theta spectral power (Peterson et al., 2018) and decreased alpha spectral power (Peterson et al., 2018; Malcolm et al., 2020) in the frontal, occipital, and cingulate regions. Subjected to obstacle appearance (Nordin et al., 2019) or visual signals on stepping position (Oliveira et al., 2018; Yokoyama et al., 2020), the participants would also exhibit significant changes in spectral power for delta, theta, and alpha bands in the supplementary motor area and the sensorimotor, premotor, and posterior parietal cortices. As mentioned above, visual intervention with a clear image could elicit changes in electrocortical dynamics associated with cerebral participation in postural balance and motor control during treadmill walking. The pattern of these changes was similar to that observed in situations of balancing tasks (Slobounov et al., 2009; Hulsdunker et al., 2015; Mierau et al., 2017), beam walking (Sipp et al., 2013), and uphill walking (Bradford et al., 2016), which was related to more active sensorimotor integration and was characterized as synchronization of the low-frequency bands and desynchronization of the alpha band.

The positive effects of visual manipulation on sensorimotor integration have provided the theoretical basis for its application in motor rehabilitation. Accordingly, in the training of balance control, increased delta-band activity over the central–frontal, central, and central parietal cortices was detected during the task of standing on an unstable surface with eyes closed, which was significantly correlated with the balance performance (Ozdemir et al., 2018). Subsequent studies on the effects of interactive visual feedback on electrocortical dynamics during treadmill training have also revealed the alpha suppression and increased activity of delta and theta bands in the sensorimotor cortex (Kaneko et al., 2021), anterior cingulate cortex, posterior parietal cortex, and inferior parietal lobe (Wagner et al., 2014; Luu et al., 2017). These results confirmed the potential significance of visual interventions with a clear image or visual deprivation in motor rehabilitation. However, these recent studies have not discussed the effects of blurred visual signals on the walking pattern and synchronous cerebral activity. Meanwhile, previous studies were mainly focused on cerebral dynamics associated with treadmill walking. During free walking, the brain needs to process online visual inputs containing information concerning the surroundings, of which the cerebral activity would be more complex. Therefore, further study should be directed to investigate the electrocortical activities in response to blurred visual inputs during the process of free walking.

The purpose of this study was to investigate the changes in gait performance and the synchronous cortical activity during free walking in response to different levels of blurred vision. The hypotheses of our study were as follows:

1. For the role of visual inputs in locomotor navigation, electrocortical activities related to visual processing would be detected during free level walking with normal vision. Due to the importance of low-frequency bands in sensorimotor integration related to vision and mobility, the activity of delta and theta bands in the occipital regions might be the main manifestation of the electrophysiological signature during walking (Peterson and Ferris, 2018; Peterson et al., 2018).
2. The mildly blurred vision could be noticed as interference with motor navigation, which would force the brain to recruit more cerebral functions to compensate the visual disturbance. The recruitment of additional cerebral function might result in the generalization of electrocortical activities in low-frequency bands from the occipital region to sensorimotor and parietal regions (Dufour et al., 2018; Peterson and Ferris, 2018; Peterson et al., 2018).
3. In circumstances of seriously blurred visual signals or visual deprivation, the brain would rely on visual working memory to complete the locomotor navigation. Based on the findings on the extraction of visual information and its integration within relevant brain regions, this process might be accompanied by the activity of low-frequency bands in the primary visual cortex (the occipital regions) (Liebe et al., 2012; Reinhart et al., 2012; Christophel et al., 2017; Sauseng et al., 2019) and synchronization of the alpha band across the sensorimotor, posterior parietal, and occipital regions (Spitzer and Blankenburg, 2012). The electrodes of Cz (corresponding to the sensorimotor area); Pz (corresponding to the parietal region); and Oz, O1, and O2 (corresponding to the occipital region) were selected as the interesting electrodes (Dufour et al., 2018; Peterson and Ferris, 2018).

## 2. Materials and methods

### 2.1. Participants

In this study, we recruited 22 healthy young volunteers with normal or corrected-to-normal vision (all men; mean age: 24.4 ± 3.9 years). All participants identified themselves as right-hand and right-foot dominant. We screened subjects for any orthopedic, cardiac, or neurological conditions and injuries. Before each experiment, the participant would be screened for symptoms of locomotor deficits by the physiotherapist in the gait laboratory. All participants provided written informed consent. Our protocol was approved by the Institutional Review Board of Peking University Third Hospital (IRB00006761-2016037).

### 2.2. Experimental design and procedures

**Figure 1:**
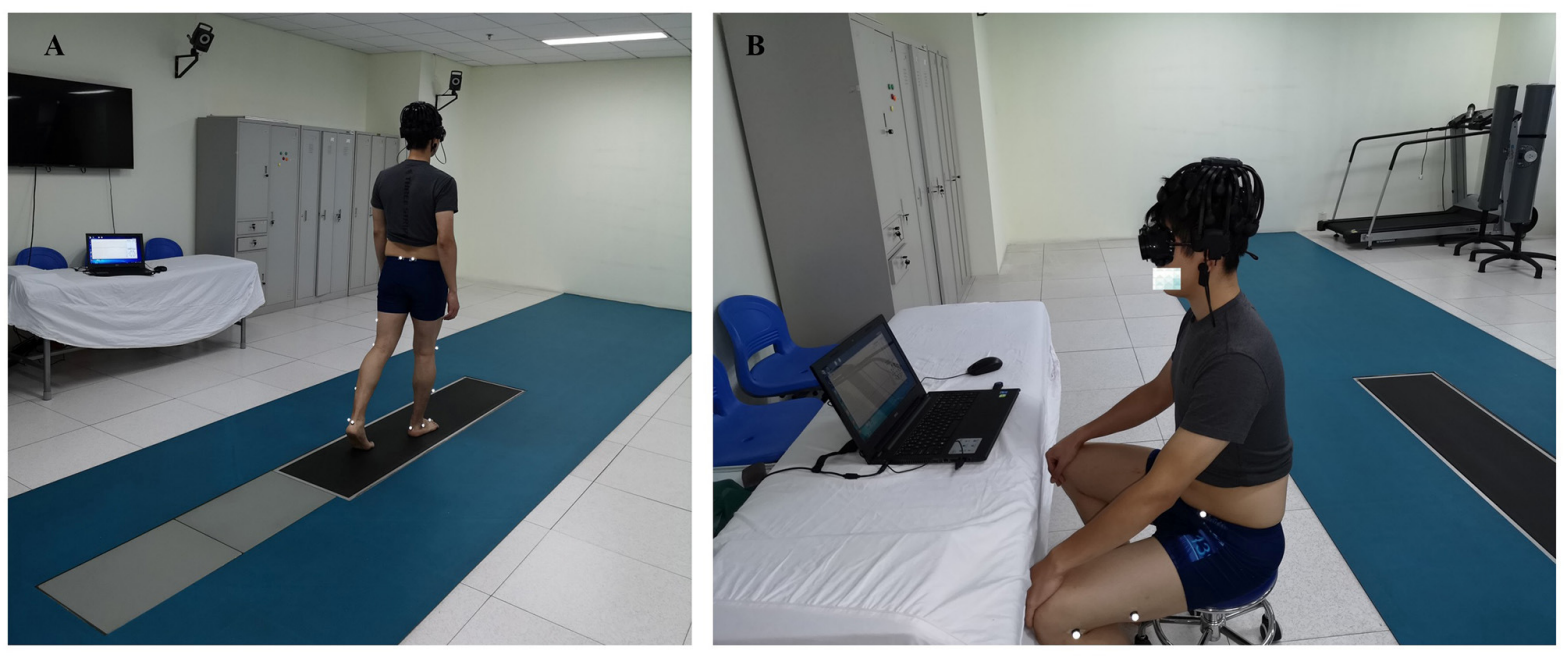
Illustrations of the experimental setup. **(A)** The gait performance was collected by the automated infrared 8-camera motion capture system with a set of markers based on the plug-in-gait model. The participant walked along the tract in the gait laboratory wearing the testing goggles. EEG signals were recorded by a wireless EEG system. **(B)** The EEG signal at the rest status was recorded for 3 min while the subject was seated on a chair with eyes closed and awake.

The schematic illustrations of the experimental setup are shown in Figure 1. All tests were taken in a professional interdisciplinary laboratory of the Department of Sports Medicine, Peking University Third Hospital, Beijing, China with the standard environment for gait testing and a quiet environment for EEG recording. The test site was illuminated by fluorescent lamps with an illuminance level of 240–250 lx at the foot ground. The floor was solid and without obstacles. A qualitative physical examination of motor function, including tone of muscle, limb strength, and angle of joint flexion and extension, was performed by the physiotherapists before the test.

There were four levels of visual status in our study. In an attempt to build a well-controlled status of blurred vision, the occlusion foil (BangerterTM, RYSER OPHTHALMOLOGIE, Switzerland) with different levels was adopted to assemble the testing goggles. According to the WHO grading criteria for low vision, the levels of occlusion foil were selected to the Snellen visual acuity of 20/60 (simulating the mildly blurred vision in the status of secondary low vision, and recorded as the condition of V0.3), 20/200 (simulating the severely blurred vision in the status of primary low vision, and recorded as the condition of V0.1), and light perception (simulating visual deprivation, recorded as the condition of V0). The participants wore goggles with both lenses covered by the same occlusion foil and completed the level walking task. Walking with the goggles, but without any occlusion foil, was recorded as the condition of V1.0. At each condition, the participants were required to complete barefoot walking blocks at the usual pace, with every block of 10 m. In each block, the participants started walking 2 m prior to the beginning of the record and finished walking 2 m after the end of the record. Five successful walking blocks were completed, and the average of the data derived from the median three walks (the second, third, and fourth walks) was analyzed. The test sequence of the four conditions (V0, V0.1, V0.3, and V1.0) was determined according to the random number in an attempt to exclude carry-over effects and learning effects. There was a 2-min interval between blocks, during which we changed the goggles. Practice walking was performed before the formal test to acquaint the participants with the environment and the procedures. The participants were uniformly instructed “After hearing the start command, please start walking from the start line marked on the floor at your usual daily speed. Please stop walking after hearing the stop command. At the end of each trial, the staff will lead you back to the starting point and prepare for the next trial. We have checked the pathway and can confirm that the path is flat and free of obstacles. During the test, the staff would accompany you on either side for your safety.” In each test, the EEG system and the motion capture system would be triggered simultaneously, in an attempt to record the EEG signals to be synchronous with gait parameters.

### 2.3. Gait measurements and data analysis

Three-dimensional coordinate data of gait performance were collected using an automated infrared 8-camera motion capture system at a sample rate of 100 Hz (Vicon MX, Oxford Metrics, UK). Two force plates at a sampling rate of 1,000 Hz (AMTI, Advanced Mechanical Technology Inc., Watertown, Massachusetts, USA) were embedded in the pathway. To track the segmental motion during walking, a set of markers were optimized based on the validated plug-in-gait model and taped to the following anatomical locations of lower limbs: the anterior and posterior superior iliac spine; the median and lateral femoral epicondyles, the malleoli, and the medial and lateral sides of the calcaneus; the frontal and lateral aspects of the thigh and the shank; the posterior part of the calcaneus; heads of the first, second, and fifth metatarsal bones; the base of the first metatarsal bone, navicular, and the hallux. The participants were asked to take the standing trial first. Then, they were asked to walk from a specified point so that one of their feet would unintentionally walk on the first force plate and the other would walk on the second force plate. A successful trial was characterized by the clear track of all the anatomical markers. The test scenario of gait measurement is illustrated in Figure 1A.

The coordinate data were filtered using a low-pass Butterworth filter at 12 Hz. Time–series data for the kinematic variables were calculated using Visual3D software (Cmotion, Germantown, MD). The heading angle is defined as the angle between the line of progression and the forward direction. It represents the deviation from a straight path. The line of progression is calculated from the sacral marker trajectory. Events of heel strike and toe-off were determined based on force platform recordings and ankle marker trajectories. From these events, step speed, stride length, double support time, stance time, and swing time were calculated. Kinematics of the hip, knee, and ankle were calculated using the Vicon Plug-in-Gait model. The parameters of hip range of motion in the sagittal plane (HROM1), the hip range of motion in the coronal plane (HROM3), hip flexion at heel strike (H0), the knee range of motion in the sagittal plane (KROM), knee flexion at heel strike (K0), knee flexion at loading response (KLR), the ankle plantar flexion at loading response (ALR), and maximal ankle dorsiflexion in stance (Adf) were selected to describe the kinematic characteristics during the gait cycle (Hallemans et al., 2009).

### 2.4. EEG recording and data analysis

The EEG data synchronous with each walking block were recorded by a wireless 30-channel dry EEG system (Cognionics Quick-30) and sampled at 1,000 Hz. The EEG signals were bandpass-filtered from 0 to 100 Hz. The Ag/AgCl electrodes were mounted on a custom-made cap according to the international 10–20 system. Electrode impedance was kept below 5 kΩ. All recorded signals were referenced to the left and right mastoids. The ground electrode was placed on the frontal head. In the tests for EEG signals during walking (Figure 1A), the subjects were asked to fix their gaze on the wall at eye level in front of them and were required to relax throughout the experiment to avoid, as far as possible, turning or bobbing their head while walking. This helped minimize artifacts arising at the electrode–skin interface. The EEG signal at the rest status (Figure 1B) was recorded for 3 min while the subject was seated on a chair with eyes closed and awake. The EEG signal at rest status was recorded previous to the EEG test synchronous with walking. Offline analysis was performed using the WinEEG software (www.mitsar-EEG.com), with a low-pass filter at 30 Hz. The independent component analysis (ICA) was employed to eliminate the blink artifacts and artifacts related to the contraction of frontal and temporal muscles or neck muscles. The EEG epochs were segmented and baseline corrected. Epochs of more than 80 μV were excluded as artifacts. The spectrum of delta (< 4 Hz), theta (between 4 and 7 Hz), and alpha (between 8 and 13 Hz) were calculated at the electrode sites of Cz, Pz, Oz, O1, and O2, respectively.

### 2.5. Statistical analysis

Statistical analyses were performed using SPSS version 22.0 (SPSS Inc., Chicago, IL, USA). The normality of the data distribution was checked by the Kolmogorov–Smirnoff–Lillefors test. Descriptive statistics for normally distributed continuous variables of gait parameters were reported as the mean ± standard deviation. The non-normally distributed variables of gait parameters were described as the median (P25, P75). We used the two-way ANOVA with a randomized block design (visual status as the group factor and individual differences as the blocking factor) to access the effects of condition (V0 vs. V0.1 vs. V0.3 vs. V1) on gait parameters with normal distribution. *Post-hoc* pairwise comparisons were performed using the least significant difference (LSD) test. In the non-parametric Friedman test performed in the analysis on non-normally distributed gait parameters, the corrected *p*-value of 0.008 (Bonferroni adjustment: *p*′ = 2*p/k* (*k* – 1), *k* = 4, *p* = 0.05) was employed in the pairwise comparisons. In the analysis of EEG data, the 5 × 5 repeated-measures ANOVA was used with the repeated factors of condition (rest vs. V0 vs. V0.1 vs. V0.3 vs. V1) and the electrode location (Cz vs. Pz vs. Oz vs. O1 vs. O2). The EEG data were reported as the mean ± standard error. Pearson correlation coefficient was calculated to investigate the association between the walking speed at different visual statuses and the spectral power of the delta, theta, and alpha band, respectively. The significance level was set at a two-sided *p*-value of < 0.05. The Greenhouse–Geisser correction was applied to the *p*-values.

## 3. Results

### 3.1. Walking pattern: a cautious gait in response to visual deprivation

There were significant main effects of visual status for the parameters of step speed [*F*_(3)_ = 10.503, *p* < 0.001] and heading angle [*F*_(3)_ = 8.000, *p* < 0.001]. *Post-hoc* tests revealed that the step speed in the visual status of V0 was significantly lower in comparison to values in the visual status of V0.1, V0.3, and V1.0 (*p* < 0.001). The participants also exhibited significantly higher heading angles at the visual status of V0 than the other three visual statuses (*p* ≤ 0.001). Analysis of stance time manifested a significant main effect of visual status [*F*_(3)_ = 6.376, *p* = 0.001]. There was a prolonged stance time in the situation of V0 in comparison with the other three statuses (*p* ≤ 0.001). Additionally, there were statistically significant differences in comparisons on the double support phase within the four visual statuses [*Z*_(3)_ = 9.257, *p* = 0.026]. The pairwise comparisons showed a significantly prolonged double support phase at the visual status of V0 in comparison with the status of V1.0 (*p* = 0.005). We could not detect the main effect of visual status for the parameter of stride length [*F*_(3)_ = 1.163, *p* = 0.331]. Similarly, the values of swing time were comparable within the four visual statuses [*Z*_(3)_ = 5.711, *p* = 0.127]. Analysis of spatial and temporal parameters for gait at different visual statuses is summarized in Table 1.

**Table 1:** Spatial and temporal parameters for gait at different visual statuses.

	**V1.0**	**V0.3**	**V0.1**	**V0**
Step speed (m/s)	1.38 ± 0.11	1.39 ± 0.11	1.39 ± 0.11	1.25 ± 0.24^*^
Heading angle (degree)	0.04 ± 0.38	0.03 ± 0.50	0.14 ± 0.45	1.73 ± 2.86^*^
Stride length (cm)	127.7 ± 19.8	128.2 ± 20.7	128.3 ± 21.3	124.4 ± 20.0
Double support time (ms)	94.5 (86.5, 106.8)	95.8 (88.3, 103.5)	95.5 (90.0, 103.5)	127.5 (90.0, 147.5)^**^
Stance time (ms)	610.4 ± 38.4	609.8 ± 29.1	603.1 ± 40.5	656.4 ± 100.2^*^
Swing time (ms)	411.2 (112.9, 424.4)	406.0 (112.4, 432.0)	406.0 (85.0, 423.0)	408.8 (79.5, 446.2)

Normally distributed continuous variables were reported as the mean ± standard deviation, and non-normally distributed variables are described as the median (P25, P75). V1.0, the status of walking with the goggles but without any occlusion foil; V0.3, the status of walking with the goggles covered by the occlusion foil with a visual acuity of 20/60; V0.1, the status of walking with the goggles covered by the occlusion foil with a visual acuity of 20/200; and V0, the status of walking with the goggles covered by the occlusion foil with only light perception.

* p < 0.05 for the two-way ANOVA in comparison with the parameter at the status of V1.0, V0.3, and V0.1.

** p < 0.008 for the non-parametric Friedman test (Bonferroni adjustment) in comparison with the parameter at the status of V1.0.

The two-way ANOVA on kinematic parameters revealed that there were significant main effects of visual status for the parameters of K0 on the left side [*F*_(3)_ = 3.828, *p* = 0.014] and HROM1 [*F*_(3)_ = 4.119, *p* = 0.010] and HROM3 [*F*_(3)_ = 12.216, *p* < 0.001] on the right side. The *post-hoc* analysis manifested that the degree of K0 on the left side was statistically increased at the visual status of V0 (*p* = 0.012, *p* = 0.023, and *p* = 0.003), while the HROM1 (*p* = 0.003, *p* = 0.006, and *p* = 0.017) and HROM3 (*p* < 0.001) on the right side was significantly lower at the visual status of V0 than that at the status of V0.1, V0.3, and V1.0. Additionally, there were statistically significant differences in comparisons on the degree of KLR on the left side [*Z*_(3)_ = 8.673, *p* = 0.034]. The pairwise comparisons showed a decrease of KLR at the status of V0 in comparison with the status of V1.0 (*p* = 0.004). The analysis of other kinematic parameters detected no main effects of visual status or statistically significant differences within the four visual conditions. The analysis of spatial–temporal and kinematic parameters of gait suggested that the parameters of gait pattern were not sensitive to blurred vision with the partial preservation of morphological visual inputs. Only in the situation of visual deprivation, the young healthy volunteers would exhibit a cautious gait pattern, characterized as a decrease in gait speed, by a greater amplitude of deviation from the right side, prolonged stance time, restricted range of motion in the hip on the right side, and increased knee flexion during stance on the left side. Comparisons on kinematic parameters for the lower extremities during walking at different visual statuses are demonstrated in Table 2.

**Table 2:** Kinematic parameters for the lower extremities during walking at different visual statuses.

	**V1.0**		**V0.3**		**V0.1**		**V0**	
	**Left**	**Right**	**Left**	**Right**	**Left**	**Right**	**Left**	**Right**
HROM1 (degree)	45.4 ± 4.3	45.6 ± 4.6	45.3 ± 3.6	45.8 ± 4.3	45.6 ± 4.1	45.9 ± 4.3	42.6 ± 10.9	44.2 ± 6.2^*^
HROM3 (degree)	12.4 ± 2.6	12.4 ± 2.7	12.0 ± 2.6	12.3 ± 2.5	12.2 ± 2.4	12.2 ± 2.8	11.2 ± 3.8	11.0 ± 2.5^*^
H0 (degree)	31.3 ± 5.3	31.0 ± 6.1	31.5 ± 5.2	31.0 ± 5.6	31.4 ± 5.1	31.0 ± 5.8	31.8 ± 5.9	31.3 ± 5.5
KROM (degree)	60.8 ± 4.6	62.4 ± 4.0	61.1 ± 5.6	62.5 ± 4.3	62.1 ± 6.3	63.2 ± 4.4	57.2 ± 14.4	62.5 ± 5.2
K0 (degree)	6.4 ± 4.6	6.3 ± 4.9	6.8 ± 5.8	6.1 ± 5.0	6.7 ± 5.4	6.4 ± 4.9	8.1 ± 6.3^*^	6.9 ± 5.2
KLR (degree)	11.6 (7.9, 14.1)	12.1 (8.8, 15.5)	11.4 (7.2, 13.2)	11.6 (9.3, 14.4)	11.6 (6.8, 13.1)	12.1 (8.8, 13.8)	9.3 (4.5, 13.8) ^**^	10.4 (8.3, 13.4)
ALR (degree)	6.7 (5.6,8.6)	15.4 (10.6, 21.7)	6.1 (5.1,7.8)	15.1 (11.9, 19.6)	6.2 (5.4,8.0)	16.4 (11.5, 19.7)	6.8 (5.2, 10.2)	16.1 (14.5, 21.3)
Adf (degree)	−1.4 (−4.5, 1.3)	−1.3 (−3.8, −0.4)	−1.5 (−4.2, 1.0)	−1.9 (−3.2, −0.5)	−1.9 (−3.8, 1.5)	−2.3 (−3.7, −0.6)	−1.4 (−4.5, 1.9)	−2.0 (−5.1, −1.0)

Normally distributed continuous variables were reported as the mean ± standard deviation, and non-normally distributed variables are described as the median (P25, P75). V1.0, the status of walking with the goggles but without any occlusion foil; V0.3, the status of walking with the goggles covered by the occlusion foil with the visual acuity of 20/60; V0.1, the status of walking with the goggles covered by the occlusion foil with the visual acuity of 20/200; and V0, the status of walking with the goggles covered by the occlusion foil with only light perception. HROM1, hip range of motion in the sagittal plane; HROM3, hip range of motion in the coronal plane; H0, hip flexion at heel strike; KROM, knee range of motion in the sagittal plane; K0, knee flexion at heel strike; KLR, knee flexion at loading response; ALR, ankle plantar flexion at loading response; Adf, maximal ankle dorsiflexion in stance.

* p < 0.05 for the two-way ANOVA in comparison with the parameter of the same side at the status of V1.0, V0.3, and V0.1.

** p < 0.008 for the non-parametric Friedman test (Bonferroni adjustment) in comparison with the parameter of the same side at the status of V1.0.

### 3.2. EEG data: changes in the distribution of low-frequency bands and power of the alpha band in response to different visual statuses

**Figure 2:**
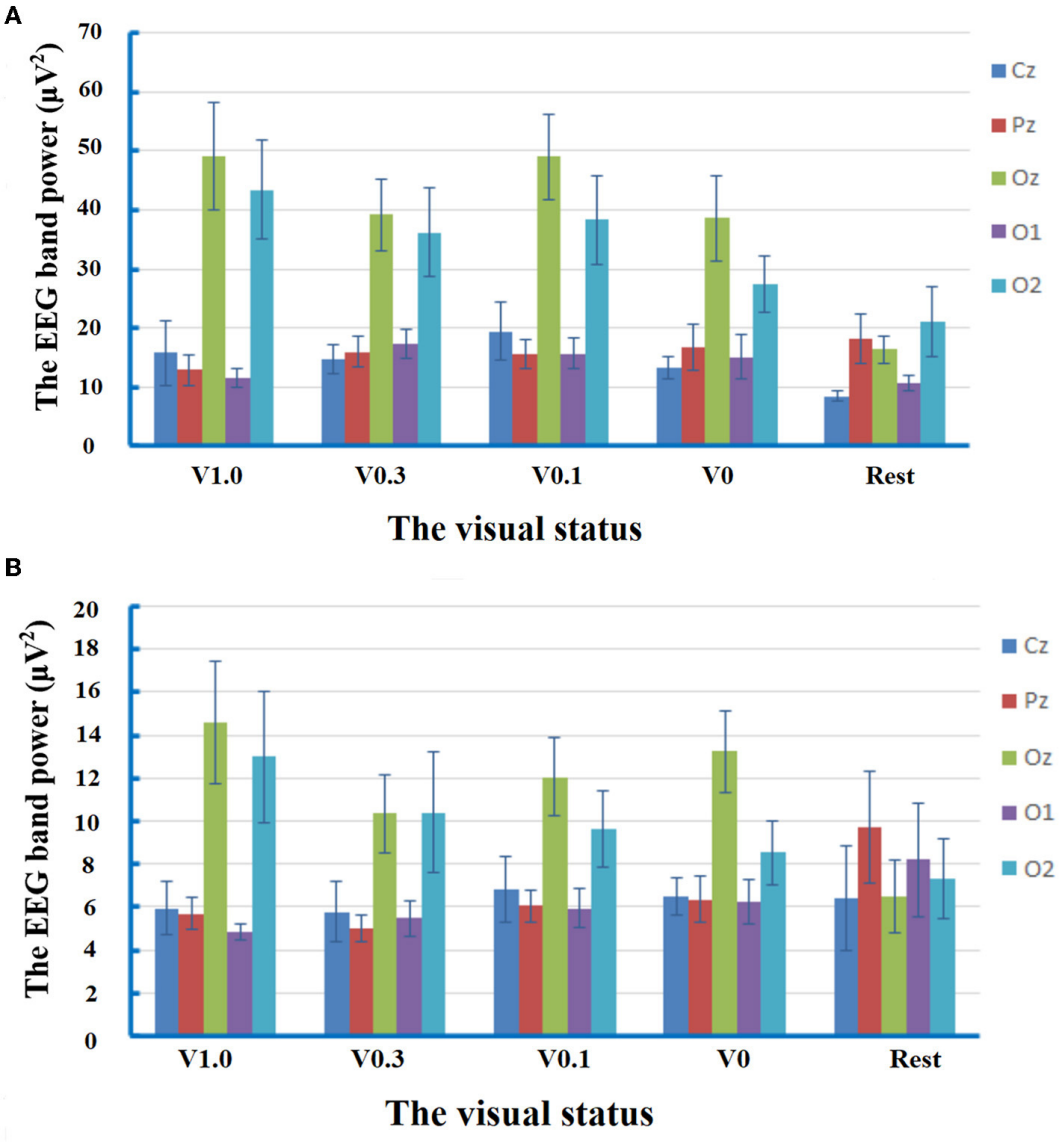
Mean values and standard errors for the band power of EEG delta frequency and theta frequency at different visual statuses. V1.0, the status of walking with the goggles but without any occlusion foil; V0.3, the status of walking with the goggles covered by the occlusion foil with the visual acuity of 20/60; V 0.1, the status of walking with the goggles covered by the occlusion foil with a visual acuity of 20/200; and V0, the status of walking with the goggles covered by the occlusion foil with only light perception. **(A)** At the visual status of V1.0, the delta activity predominant in the occipital regions with higher spectral power at the electrodes of Oz and O2 in comparison to that at the electrodes of Cz (*p* = 0.013 and *p* = 0.033), Pz (*p* = 0.015 and *p* = 0.030), and O1 (*p* = 0.009 and *p* = 0.017). At the visual status of V0.3, the predominant distribution of delta activity was attenuated with higher power only at the electrode of Oz in comparison with that at the electrode of Cz (*p* = 0.049). In response to severe visual restriction, the distribution of delta activity in occipital regions was observed again. At the visual status of V0.1, the spectral power at the electrodes of Oz was statistically higher than Cz (*p* = 0.004), Pz (*p* = 0.007), and O1 (*p* = 0.004), while the power at O2 was higher than Pz (*P*=0.035) and O1 (*P*=0.045). At the status of V0, there was higher spectral power at the electrodes of Oz and O2 in comparison to that at the electrodes of Cz (*p* = 0.013 and *p* = 0.022), Pz (*p* = 0.047 and *p* = 0.035), and O1 (*p* = 0.021 and *p* = 0.021). At the rest status, the spectral power of the delta band at the electrode of Oz was statistically higher than that at Cz (*p* = 0.038). **(B)** At the visual status of V1.0, the spectral power of the theta band at Oz was higher than that at the electrodes of Cz (*p* = 0.044) and O1 (*p* = 0.044). At the visual status of V0.3, the spectral power was compatible within the five interested electrodes. At the visual status of V0.1, the trend of predominant distribution of theta activity in the central occipital region was observed again with higher spectral power at the electrode of Oz in comparison with that at Cz (*p* = 0.010). At the status of V0, the predominant distribution of theta activity was detected in the central occipital region with higher spectral power at the electrode of Oz in comparison to that at the electrodes of Cz (*p* = 0.006), Pz (*p* = 0.016), and O1 (*p* = 0.009). At the rest status, the spectral power was compatible within the five interested electrodes.

The repeated-measures ANOVA for EEG band power of delta frequency yielded no significant main effect of condition [*F*_(2.812)_ =2.881, *p* = 0.051, partial η^2^ = 0.171]. There was a significant main effect of electrode location [*F*_(2.236)_ = 24.217, *p* < 0.001, partial η^2^ = 0.634]. The *post-hoc* comparisons manifested that the spectral power of the delta band recorded at the electrodes of Oz (38.48 ± 3.64 μV^2^) and O2 (33.29 ± 3.79 μV^2^) was statistically higher than that at the electrodes of Cz (14.35 ± 1.88 μV^2^, *p* < 0.001, respectively), Pz (15.89 ± 1.78 μV^2^, *p* = 0.002 and *p* = 0.010, respectively), and O1 (14.04 ± 1.36 μV^2^, *p* < 0.001 and *p* = 0.002, respectively). The interaction of condition × electrode location [*F*_(5.797)_ = 2.462, *p* = 0.032, partial η^2^ = 0.150] was also significant. The subsequent multiple comparison tests manifested that, in comparison with the status of rest (16.36 ± 2.34 μV^2^), the delta activity at the electrode of Oz increased significantly during walking at the visual status of V1.0 (49.15 ± 9.06 μV^2^, *p* = 0.013), V0.3 (39.22 ± 6.00 μV^2^, *p* = 0.049), and V0.1 (49.01 ± 7.35 μV^2^, *p* = 0.015). At the visual status of V1.0, analysis of spectral power at the interested electrodes revealed that the delta activity was significantly higher at the electrodes of Oz (49.15 ± 9.06 μV^2^) and O2 (43.47 ± 8.27 μV^2^) compared to that at the electrodes of Cz (15.77 ± 5.46 μV^2^, *p* = 0.013 and *p* = 0.033, respectively), Pz (12.90 ± 2.65 μV^2^, *p* = 0.015 and *p* = 0.030, respectively), and O1 (11.4 7 ± 1.66 μV^2^, *p* = 0.009 and *p* = 0.017, respectively). At the visual status of V0.3, the delta activity was only significantly higher at the electrode of Oz (39.22 ± 6.00 μV^2^) in comparison with that at the electrode of Cz (14.73 ± 2.56 μV^2^, *p* = 0.049). Interestingly, at the visual status of V0.1, the trend of predominant distribution of delta activity in the occipital regions was observed again, which was manifested as the statistically higher spectral power at the electrodes of Oz (49.01 ± 7.35 μV^2^) vs. Cz (19.46 ± 4.99 μV^2^, *p* = 0.004), Pz (15.62 ± 2.48 μV^2^, *p* = 0.007), O1 (15.67 ± 2.67 μV^2^, *p* = 0.004); and O2 (38.32±7.7.56 μV^2^) vs. Pz (*p* = 0.035), O1 (*p* = 0.045). Moreover, at the status of V0, the distribution of delta activity was also predominant in the occipital regions with higher spectral power at the electrodes of Oz (38.66 ± 7.25 μV^2^) and O2 (27.49 ± 4.81 μV^2^) in comparison to that at the electrodes of Cz (13.29 ± 1.85 μV^2^, *p* = 0.013 and *p* = 0.022, respectively), Pz (16.73 ± 3.89 μV^2^, *p* = 0.047 and *p* = 0.035, respectively), and O1 (15.02 ± 3.76 μV^2^, *p* = 0.021 and *p* = 0.021, respectively). At the rest statuses, the spectral power of the delta band at the electrode of Oz (16.36 ± 2.34 μV^2^) was statistically higher than that at Cz (8.50 ± 0.88 μV^2^, *p* = 0.038). The results of the analysis on the delta band suggested that there were cerebral activities related to the delta band in the process of free level walking. The response of delta activity in circumstances to different visual statuses during locomotor navigation was mainly characterized by the changes in its predominant distribution in occipital regions. The results of the analysis on delta band power are shown in Figure 2A.

The repeated-measures ANOVA for EEG band power of theta frequency revealed no significant main effect of condition [*F*_(2.832)_ = 0.217, *p* = 0.874, partial η^2^ = 0.015]. There was a significant main effect of electrode location [*F*_(2.424)_ = 9.396, *p* < 0.001, partial η^2^ = 0.402]. The *post-hoc* comparisons manifested that the spectral power of theta band measured at the electrodes of Oz (11.34 ± 1.07 μV^2^) was statistically higher than that at the electrodes of Cz (6.28 ± 0.80 μV^2^, *p* = 0.011), Pz (6.56 ± 0.58 μV^2^, *p* = 0.009), and O1 (6.13 ± 0.69 μV^2^, *p* = 0.003). The interaction of condition × electrode location [*F*_(5.171)_ = 2.389, *p* = 0.044, partial η^2^ = 0.146] was significant. The subsequent multiple comparison tests manifested that, at the visual status of V1.0, the spectral power at Oz (14.58 ± 2.84 μV^2^) was significantly higher than that at the electrodes of Cz (5.91 ± 1.24 μV^2^, *p* = 0.044) and O1 (4.83 ± 0.35 μV^2^, *p* = 0.044). At the visual status of V0.3, the spectral power was compatible with the five interested electrodes. At the visual status of V0.1, there was statistically higher spectral power at the electrode of Oz (12.06 ± 1.84 μV^2^) in comparison with that at Cz (6.82 ± 1.50 μV^2^, *p* = 0.010). At the status of V0, the distribution of theta activity was predominant in the central occipital region with higher spectral power at the electrode of Oz (13.23 ± 1.91 μV^2^) in comparison to that at the electrodes of Cz (6.49 ± 0.86 μV^2^, *p* = 0.006), Pz (6.35 ± 1.06 μV^2^, *p* = 0.016), and O1 (6.23 ± 1.02 μV^2^, *p* = 0.009). At the rest status, the spectral power was compatible with the five interested electrodes. These results manifested the predominant distribution of theta activity in the central occipital region in response to visual deprivation. Data of theta band power are exhibited in Figure 2B.

In an analysis of the spectral power of the alpha band, the repeated-measures ANOVA revealed a significant main effect of condition [*F*_(2.362)_ = 10.454, *p* < 0.001, partial η^2^ = 0.427]. The *post-hoc* comparisons manifested that the spectral power of the alpha band at the visual status of V0 (16.09 ± 1.61 μV^2^) was significantly higher than that recorded at the rest status (10.07 ± 1.52 μV^2^, *p* = 0.024) and visual status of V1.0 (7.83 ± 0.76 μV^2^, *p* = 0.006), V0.3 (8.47 ± 0.90 μV^2^, *p* = 0.011), and V0.1 (8.47 ± 0.77 μV^2^, *p* = 0.001). The main effect of electrode location was not statistically significant [*F*_(1.721)_ = 2.146, *p* = 0.144, partial η^2^ = 0.133]. The interaction of condition × electrode location did not reach the significant level [*F*_(3.907)_ = 2.496, *p* = 0.055, partial η^2^ = 0.151]. These results suggested that, during level walking, there was a synchronization of the alpha-band activity across the interested electrodes in response to visual deprivation.

The Pearson correlation analysis, at the status of V0.3, statistically significant correlations were observed between the walking speed and the spectral power of the delta band at the electrode of Cz (*p* = 0.029, *r* = −0.562) and between the walking speed and the spectral power of theta band at the electrodes of Pz (*p* = 0.005, *r* = 0.681) and O1 (*p* = 0.013, *r* = 0.626). These potential correlations detected at low-frequency bands at the status of V0.3 manifested that the brain would recruit more cerebral regions to complete the sensorimotor integration. Meanwhile, at the status of V0.1, statistically significant correlations were observed between the walking speed and the spectral power of the alpha band at the electrodes of Pz (*p* = 0.006, *r* = −0.669) and O1 (*p* = 0.023, *r* = −0.582), indicating increased cortical involvement in motor control. The correlation analysis of data from V1.0 and V0 revealed no significant results (*p* > 0.05). The results of Pearson correlation analysis were manifested in Supplementary Table S1.

## 4. Discussion

The changes in walking and cerebral activity during treadmill walking in response to visual intervention have been previously investigated. However, the effects of blurred vision with different levels on gait performance and cerebral function synchronous with free walking have not yet been discussed. The purpose of this study was to evaluate how the simulated blurred vision with different levels would affect the gait pattern and electrocortical activities during free level walking in young healthy volunteers. Our results demonstrated that, in free level walking with normal vision, there were cerebral activities related to visual processing, characterized as activities of delta and theta bands in the occipital region. The moderately blurred visual inputs would attenuate the predominance of delta- and theta-band activities in the occipital region. In the circumstances of severely blurred vision and visual deprivation with only light perception, the predominant distribution of low-frequency bands would emerge again, while the cautious gait pattern was only observed at the status of visual deprivation with retention of light perception. There was also synchronization of alpha-band activity across the sensorimotor, posterior parietal, and occipital regions during free level walking at the visual status of only light perception. The present study's results suggested that the mildly blurred visual inputs would elicit the generalization of delta- and theta-band activities from the occipital region to other cerebral regions, indicating the recruitment of more cerebral regions to maintain a normal walking pattern. During walking with visual inputs of only light perception, the visual-related locomotor navigation would rely on the recall of visual working memory, which was characterized by activities of low-frequency bands in occipital regions and synchronization of the alpha band across sensorimotor, posterior parietal, and occipital regions. The severe visual interference such as blurred visual signal delivered by occlusion foil targeting the Snellen visual acuity of 20/200 might be the threshold to trigger the shift of different mechanisms of sensorimotor integration.

### 4.1. A pattern of cautious gait in response to visual deprivation

According to the analysis of spatial–temporal parameters of gait, the complete deprivation of visual inputs about the morphological signal (V0) could result in a cautious gait pattern. The main changes were manifested as a decrease in gait speed, greater amplitude of deviation from the right ahead, and prolonged stance time. The lower walking speed (Hallemans et al., 2010; Ayaki et al., 2015) and greater amplitude of deviation from the right ahead (Hallemans et al., 2010) have also been reported in previous studies on changes in gait when sighted individuals were blindfolded temporarily. The slower walking could be noticed as a typical feature of cautious gait for which it would allow for more time for hepatic exploration. In individuals with normal vision, the orientation of locomotion mostly relied on the information delivered by visual signals, the loss of which would result in difficulties in control of direction and deviation from the right ahead. The prolonged stance time implied that the individuals would extend the duration of foot-to-ground contact to maintain gait stability.

The analysis of kinematic parameters showed that the changes in joint motion underlying the cautious gait were mainly related to the range of motion in the hip on the right side and the knee on the left side. It had been reported that temporary blindfolding in individuals with normal vision would result in a decrease in the hip range of motion in the sagittal plane (Hallemans et al., 2010), which was consistent with our results. The hip was the proximal element in the kinematic chain of the lower extremities with significant importance in gait stability (Arumukhom Revi et al., 2021). The decreased range of motion in the hip indicated that, in the absence of visual navigation, an essential part of the motor adjustment was the limited movement in the supporting joint on the dynamic side to maintain stability. In the process of walking, the knee was the direct effector of locomotion (Dounskaia, 2010), which would load the body weight. The increased range of flexion motion in the left knee during stance indicated that the knee on the supporting side would act as an elastic buffer to compensate the instability in the circumstance of visual deprivation.

### 4.2. The involvement of low-frequency bands in locomotor control during free walking

The results of our study manifested that there was a predominant distribution of the delta band in occipital regions during free walking even in the situation with normal visual inputs (V1.0). Previous studies concerning cerebral activities during locomotion have found that the information for decoding lower limb kinematics during treadmill walking is contained in activities of the delta band (Bradberry et al., 2011; Presacco et al., 2011). In a subsequent study, it has been reported that pre-movement delta band amplitude could be used to decode locomotive intents about posture transitions (Bulea et al., 2014). Investigation of cortical correlates of muscle activation during walking has revealed that the activation of locomotor muscle synergies could be decoded from delta oscillation (Yokoyama et al., 2019). In an analysis of the spectral power of theta band, we also found a predominant distribution in the central occipital region during sighted walking, which was consistent with results in a previous study about the electrocortical dynamics synchronous with free walking in the real world (Pizzamiglio et al., 2018). The predominance of low-frequency activities in the occipital regions provided auxiliary evidence for the involvement of sensorimotor integration related to visual signals in locomotor navigation, even in the process of level walking with normal sight.

### 4.3. Generalization of low-frequency bands in response to mildly blurred vision during locomotion

According to our results, the moderate disturbance in the clarity of visual signals (V0.3) could attenuate the predominance of delta and theta bands in the occipital regions, indicating the generalization of low-frequency activities and recruitment of more cerebral functions. In a study on the cerebral response to visual cues on an obstacle during treadmill walking, a significant increase in spectral power for the delta band in the supplementary motor area, premotor, and posterior parietal cortex has been observed (Nordin et al., 2019). In human treadmill walking with brain-computer interface control, significantly increased activity in the delta frequency in the anterior cingulate cortex has been recorded in the process of viewing on walking avatar (Luu et al., 2017). The increased power of theta frequency in the anterior cingulate and sensorimotor cortices and the posterior parietal lobe has also been reported to be associated with treadmill walking with visual indicators for foot placement (Yokoyama et al., 2020) or a transient view of rotation (Peterson and Ferris, 2018; Peterson et al., 2018). These previous studies confirmed the role of low-frequency oscillations in cerebral activities during demanding walking with visual perturbations, the characteristic of which was the recruitment of multiple brain regions. In circumstances of moderately blurred visual inputs, the brain might need to extract meaningful visual cues from the background of blurred signals and maintain motor navigation. Thus, the brain needs to mobilize more functional regions related to cognition, mistake monitoring, and motor control, the recruitment of which resulted in the generalization of low-frequency bands. The potential correlation detected between the walking speed and spectral power at the electrodes of Pz and O1 provided further evidence for the hypothesis that the brain would recruit more cerebral regions to complete the sensorimotor integration in response to mildly blurred visual signals.

### 4.4. Electrocortical activities related to visual working memory during walking with severely blurred vision and visual deprivation

In the situation of severely blurred vision and visual deprivation with retention of light perception (V0.1 and V0), we detected the predominance of delta- and theta-band activities in the occipital regions during level walking, which was similar to the pattern observed in walking with normal sight. According to a previous study, increased activations in the delta band over central–frontal, central, and central parietal cortices were observed in challenging postural control with eyes closed (Ozdemir et al., 2018). Treadmill walking with eyes closed could also induce significantly increased theta desynchronization during stance (Oliveira et al., 2017). Moreover, emerging studies on mechanisms of visual working memory have revealed that the theta-band synchronization could coordinate the communications within cerebral regions and might contribute to the maintenance of visual short-term memories (Liebe et al., 2012; Reinhart et al., 2012; Christophel et al., 2017; Sauseng et al., 2019). During the 10-m level walking in the present study, the participants needed to rely on retrieval of visual memory to navigate locomotion. Therefore, in accordance with the existing findings, our results provided further evidence for the importance of cerebral activities related to low-frequency bands in the process of level walking without enough visual inputs, which might be related to the recall of visual working memory.

The data in our study manifested correlations between the spectral power of the alpha band at the electrode of Pz and O1 at the status of severely blurred visual inputs (V0.1). According to previous studies, significant suppression of alpha band has been observed in treadmill walking with visual signals for the position of the foot (Yokoyama et al., 2020), symbol of obstacles (Nordin et al., 2019), virtual reality feedback (Wagner et al., 2014), and treadmill walking with transient view perturbations (Malcolm et al., 2018; Peterson and Ferris, 2018; Peterson et al., 2018) or eyes closed (Oliveira et al., 2017). The potential negative correlations detected in the present study could be noticed as the manifestation of increased cortical involvement in motor control. Moreover, there was a synchronization of the alpha band during level walking with visual deprivation. A reasonable explanation for the changes in alpha activity might be the need for visual working memory to accomplish free level walking, characteristic of which was the topographical distribution of alpha power activity (Spitzer and Blankenburg, 2012). Thus, our results indicated that the activity of the alpha band across the sensorimotor and occipital-parietal cortices might play an important role in the extraction of visual working memory for locomotor navigation.

### 4.5. Limitations

There are several limitations that should be considered in the present study. Restricted by the relatively small number of channels in the wireless EEG system, we could not perform the analysis of the independent components to identify neural components related to the changes in cerebral activities. In the future study, an EEG system with multichannel arrays should be considered in an attempt to reveal intensive information concerning the neural components that participated in cerebral reaction to blurred vision during walking. Meanwhile, the present study analyzed the changes of power corresponding to the whole time of each walking test. Future works should focus on more detailed assessments of brain activity in response to changes in the visual status within different phases of the gait cycle, in which time-locked analysis on event-related potentials would be employed. Both attention and distance to the fixation point were important factors affecting cerebral activity related to vision. Therefore, the subsequent study would be designed to evaluate the effects of these factors on EEG data during walking with different visual statuses. We found the changes in cerebral activities in response to blurred visual inputs with different levels during free walking, while the effects of blurred vision on cerebral involvement during motor rehabilitation have not been assessed. In subsequent studies, the specific level of blurred visual inputs with optimal efficiency in visually assisted locomotor rehabilitation would be explored.

## 5. Conclusion

The results of the present study suggested that there were differences in the effects of blurred visual input with different levels on the cerebral activity during free level walking. Mildly blurred visual inputs could attenuate the predominant distribution of delta and theta bands in occipital regions, while they have no significant effects on gait performance. The generalization of low-frequency bands indicated that the brain needed to recruit more cerebral functions to resist the interference of blurred images on sensorimotor integration and maintain effective locomotor navigation. Severe visual restriction and visual deprivation with the retention of light perception would both elicit the predominant distribution of low-frequency bands in the occipital regions, while only visual deprivation with the retention of light perception would cause cautious gait and an increase of spectral power of the alpha band. These changes in EEG signals suggested that, in circumstances with no effective input of visual signal, the employment of visual working memory would play an important role in locomotor navigation. The threshold to initiate the shift of different mechanisms of sensorimotor integration might be the visual status that is as blurred as the level of Snellen visual acuity of 20/200. The combination of visual feedback and visual restriction with different levels might provide an alternative to exploring brain-computer interfaces with improved efficiency in motor rehabilitation.

## Data availability statement

The raw data supporting the conclusions of this article will be made available by the authors, without undue reservation.

## Ethics statement

The studies involving human participants were reviewed and approved by the Institutional Review Board of Peking University Third Hospital. The patients/participants provided their written informed consent to participate in this study.

## Author contributions

MA contributed to the study concept and design, data acquisition, data analysis and interpretation, statistical analysis, and drafting and revising the manuscript. SR contributed to gait parameters extraction and analysis. YY contributed to gait parameters acquisition and analysis. HH contributed to gait parameters acquisition. XM contributed to EEG data acquisition and data analysis. YA contributed to the study concept and design, supervision, data interpretation, and revising of the manuscript. WW contributed to the study concept and design, supervision and data interpretation. All authors agree to be accountable for the content of the work.
