# Supplementary material for: The effects of blurred visual inputs with different levels on the cerebral activity during free level walking
Source: Front Neurosci. 2023 Apr 17;17:1151799. doi: 10.3389/fnins.2023.1151799 (PMC10149992; doi:10.3389/fnins.2023.1151799)
Supplement: Supplementary file 1 [file Table_1.docx]

Supplementary Material

The effects of blurred visual input with different levels on the cerebral activity during free level walking

# Supplementary Table

Results of Pearson correlation analysis on walking speed and spectral power of delta, theta and alpha band at different visual status

| Electrode | Delta band | |  | Theta band | |  | Alpha band | |
| --- | --- | --- | --- | --- | --- | --- | --- | --- |
|  | *r* | *P* values |  | *r* | *P* values |  | *r* | *P* values |
| **V0** |  |  |  |  |  |  |  |  |
| Cz | -0.314 | 0.254 |  | -0.150 | 0.594 |  | -0.231 | 0.407 |
| Pz | 0.143 | 0.612 |  | -0.010 | 0.971 |  | -0.357 | 0.191 |
| Oz | -0.294 | 0.287 |  | -0.382 | 0.160 |  | -0.293 | 0.290 |
| O1 | 0.203 | 0.467 |  | 0.063 | 0.823 |  | -0.199 | 0.478 |
| O2 | 0.031 | 0.912 |  | 0.006 | 0.982 |  | 0.179 | 0.523 |
| **V0.1** |  |  |  |  |  |  |  |  |
| Cz | -0.071 | 0.801 |  | -0.107 | 0.704 |  | 0.086 | 0.759 |
| Pz | -0.085 | 0.765 |  | -0.233 | 0.404 |  | -0.669 | 0.006^*^ |
| Oz | -0.235 | 0.398 |  | -0.160 | 0.568 |  | 0.069 | 0.806 |
| O1 | -0.120 | 0.671 |  | -0.163 | 0.561 |  | -0.582 | 0.023^*^ |
| O2 | 0.016 | 0.955 |  | 0.002 | 0.993 |  | -0.251 | 0.367 |
| **V0.3** |  |  |  |  |  |  |  |  |
| Cz | -0.562 | 0.029^*^ |  | -0.384 | 0.157 |  | -0.149 | 0.596 |
| Pz | -0.126 | 0.655 |  | 0.681 | 0.005^*^ |  | 0.279 | 0.315 |
| Oz | 0.155 | 0.582 |  | 0.346 | 0.206 |  | 0.319 | 0.247 |
| O1 | 0.017 | 0.952 |  | 0.626 | 0.013^*^ |  | 0.296 | 0.284 |
| O2 | 0.327 | 0.234 |  | 0.137 | 0.626 |  | 0.099 | 0.726 |
| **V1.0** |  |  |  |  |  |  |  |  |
| Cz | 0.157 | 0.577 |  | 0.178 | 0.526 |  | 0.295 | 0.285 |
| Pz | 0.063 | 0.822 |  | 0.273 | 0.324 |  | 0.001 | 0.998 |
| Oz | -0.378 | 0.165 |  | -0.366 | 0.180 |  | -0.432 | 0.107 |
| O1 | 0.081 | 0.774 |  | 0.409 | 0.131 |  | 0.140 | 0.618 |
| O2 | 0.146 | 0.605 |  | 0.158 | 0.574 |  | 0.150 | 0.595 |

^*^, statistically significant correlation in the Pearson correlation analysis.
